# Comparison between signet-ring cell carcinoma and non-signet-ring cell carcinoma of the stomach: clinicopathological parameters, epidemiological data, outcome, and prognosis—a cohort study of 123 patients from a non-endemic country

**DOI:** 10.1186/s12957-022-02699-8

**Published:** 2022-07-20

**Authors:** Haithem Zaafouri, Raja Jouini, Nizar Khedhiri, Fatma Khanchel, Mona Cherif, Meryam Mesbahi, Aziz Daghmouri, Wiem Mahmoudi, Soumaya Akremi, Meriam Sabbah, Yazid Benzarti, Dhafer Hadded, Dalila Gargouri, Mourad Ben Bader, Anis Ben Maamer

**Affiliations:** 1 Department of General Surgery, Habib Thameur Hospital, Tunis, Tunisia; 2 Department of Cytopathology, Habib Thameur Hospital, Tunis, Tunisia; 3 Department of Anesthesiology, Habib Thameur Hospital, Tunis, Tunisia; 4 Department of Gastroenterology, Habib Thameur Hospital, Tunis, Tunisia

**Keywords:** Gastric cancer, Adenocarcinoma, Signet-ring cell carcinoma, Non-signet-ring cell carcinoma, Prognosis

## Abstract

**Background:**

Signet-ring cell carcinoma of the stomach (SRCC) is a particular gastric cancer entity. Its incidence is increasing. Its diagnosis is pathological; it corresponds to adenocarcinoma with a majority of signet-ring cells component (> 50%).

These histological features give it its aggressiveness characteristics. This has repercussions on the prognostic level and implications for the alternatives of therapy, especially since some authors suggest a potential chemoresistance.

This survey aimed to identify the epidemiological, pathological, therapeutic, and prognostic characteristics of SRCC as a separate disease entity.

**Methods:**

This was a retrospective study of 123 patients admitted for gastric adenocarcinoma to Habib Thameur Hospital in Tunis over 11 years from January 2006 to December 2016. A comparative study was performed between 2 groups: the SRCC group with 62 patients and the non-SRCC (non-signet-ring cell carcinoma of the stomach) with 61 patients.

**Results:**

The prevalence of SRCC in our series was 50%. SRCC affected significantly younger patients (55 vs 62 years; *p* = 0.004). The infiltrative character was more common in SRCC tumors (30.6 vs 14.8%; *p* = 0.060), whereas the budding character was more often noted in non-SRCC tumors (78.7 vs 58.1%; *p* = 0.039). There was no significant difference in tumor localization between both groups. Linitis plastica was noted in 14 patients with SRCC against a single patient with non-SRCC (*p* = 0.001). The tumor size was more important in the non-SRCC group (6.84 vs 6.39 cm; *p* = 0.551). Peritoneal carcinomatosis was noted in 4.3% of cases in the SRCC group versus 2.2% of cases in the NSRCC group (*p* = 0.570). Total gastrectomy was more often performed in the SRCC group (87 vs 56%; *p* = 0.001). Resection was more often curative in the non-SRCC group (84.4 vs 78.3%; *p* = 0.063). Postoperative chemotherapy was more commonly indicated in the SRCC group (67.4 vs 53.3%; *p* = 0.339). Tumor recurrence was more common in the non-SRCC group (35.7 vs 32%; *p* = 0.776). The most common type of recurrence was peritoneal carcinomatosis in the SRCC group (62.5%) and hepatic metastasis in the non-SRCC group (60%; *p* = 0.096). The overall 5-year survival in the SRCC group was lower than in the non-SRCC group, with no statistically significant difference (47.1 vs 51.5%; *p* = 0.715). The overall survival was more important for SRCC in early cancer (100 vs 80%; *p* = 0.408), whereas it was higher for non-SRCC in advanced cancer (48.1 vs 41.9%; *p* = 0.635).

**Conclusion:**

Apart from its epidemiological and pathological features, SRCC seems to have a worse prognosis. Indeed, it is diagnosed at a more advanced stage and has a worse prognosis in advanced cancer than non-SRCC. It is therefore to be considered as a particular entity of gastric adenocarcinoma requiring a specific therapeutic protocol where the place of chemotherapy remains to be more investigated.

## Introduction

Gastric cancer is considered one of the most common malignancies worldwide with more than 700,000 deaths every year [1]. A higher incidence of this type of cancer is shown in East Asian countries [2]. During the last two decades, gastric cancer prognosis did not improve despite the advances in diagnosis and new treatment options, and the 5-year-survival rate did not exceed 30% [3, 4]. Additionally, as a subtype of gastric adenocarcinoma, the proportion of signet-ring cell carcinoma (SRCC) has increased [5]. It is defined histologically according to the World Health Organization (WHO) as an adenocarcinoma in which more than 50% of tumor cells are signet-ring cells (containing abundant intracytoplasmic mucin which pushed the nucleus to the periphery) [6]. Besides, according to other histological classifications, it is an “infiltrative type” according to Ming, “diffuse type” in Lauren’s classification, and “undifferentiated type” in Sugano’s classification [7–9].

Several studies have reported that compared to non-signet-ring cell carcinoma (non-SRCC), SRCC patients were younger with a higher incidence of lymph nodes metastasis and distal metastasis [10]. In addition to that, SRCC prognosis is still a controversial subject. For example, Gronnier and Otsuji found that SRCC had better survival outcomes [11, 12]. However, according to Li, Kim, and Yokota, SRCC had a similar or worse survival prognosis compared to non-SRCC patients [13–15]. Recently, a systematic review and meta-analysis by Zhang et al. including 36 studies showed that for patients with advanced stage, SRCC was a negative prognostic factor [16]. However, concerning non-endemic countries, only four studies are available (from France and Belgium) with small sample sizes and further limitations.

So, in the present study, we aimed to identify the epidemiological, pathological, therapeutic, and prognostic characteristics of this type of gastric cancer and to examine the survival prognosis of SRCC compared to non-SRCC in an African non-endemic country.

## Methods

One-hundred and twenty-three consecutive patients diagnosed with gastric cancer admitted to the department of visceral surgery of Habib Thameur Hospital in Tunisia during the period going from January 2006 to December 2016 were included in this trial.

### Inclusion criteria

Our study included all patients treated for adenocarcinoma of the stomach independently of the site of the tumor and of the fact that whether or not the patient had undergone surgery for that.

### Exclusion criteria

We did not include in this study tumors of the adjacent organs with gastric involvement, benign tumors of the stomach, histotypes of cancers other than adenocarcinomas, and adenocarcinoma of the cardia.

We extracted from their medical records information on demographic data (age and sex), time and circumstances of diagnosis, site of tumor at diagnosis (fundus, body, or antrum), and stage of the tumor (according to the 7th edition of the American Joint Committee on Cancer staging system), further special investigations, histotype and classification of the tumor, the surgical procedure used, chemotherapy (if applicable), and overall survival with a 5-year follow-up.

To define the histological type, experienced pathologists examined all the tissues using the WHO classification (Lauren’s and Sugano’s classifications were not routinely used). Surgical procedures including curative or palliative operations were done by experienced surgeons. Overall survival was defined as the time from diagnosis to the last day of follow-up (maximum 5 years) or the day of death (all causes of death). The number of patients lost to follow-up did not exceed 5%.

**Fig. 1:**
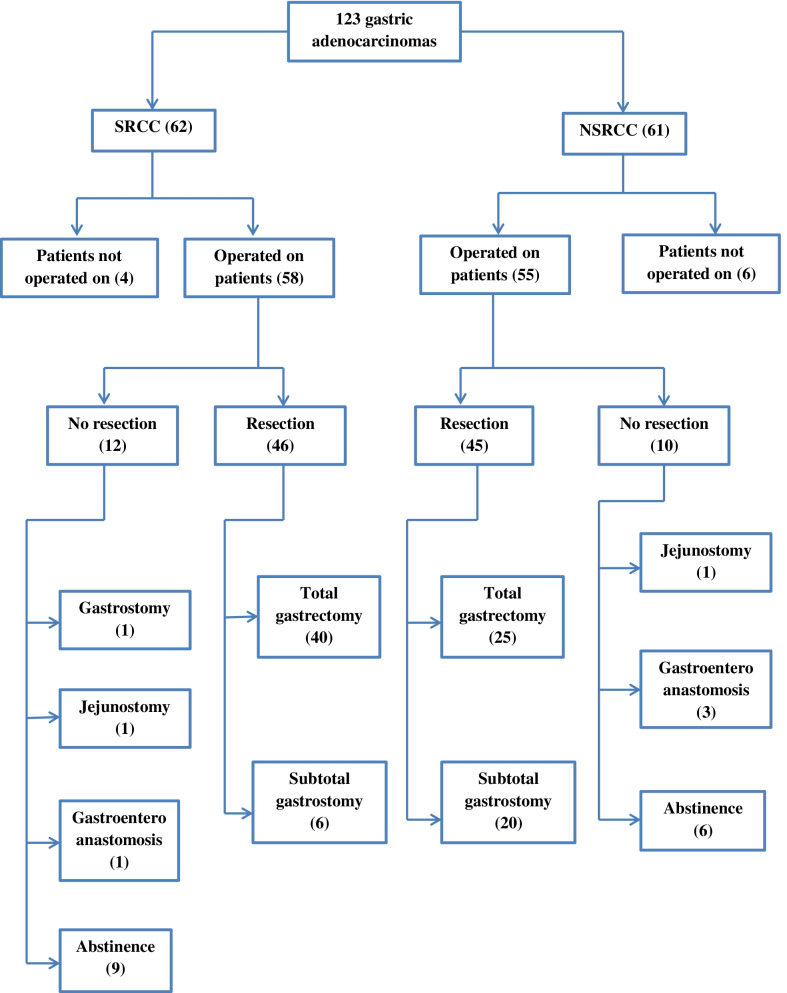
Algorithm of management of all the 123 patients

The study was approved by the Ethics Committee of Habib Thameur Hospital of Tunisia. The algorithm of management of all the 123 patients is summarized in Fig. 1.

## Results

Of the 123 patients included in this study, 62 (50.4%) had SRCC and 61 (49.6%) had non-SRCC (Table 1). SRCC patients were significantly younger than non-SRCC patients (54.9 vs 62.5 years, *p* = 0.004), and females were more likely to have SRCC than males with a male-to-female ratio in SRCC of 0.63 and non-SRCC 2.05 (*p* = 0.002).

**Table 1:** Patient characteristics

	SRCC group (***n*** = 62)	Non-SRCC group (***n*** = 61)	***p***
**Age (years)**	54.88 ± 12.59	62.47 ± 15.48	**0.004**
**Sex**			
**Female**	38 (61.3%)	20 (32.8%)	**0.002**
**Male**	24 (38.7%)	41 (67.2%)	
**Family history of cancer**			
**Gastric cancer**	0	1	0.65
**Digestive cancer**	1	0	0.45
**Smoking**	18 (29%)	20 (32.8%)	NS
**Gastric ulcer**	3 (4.8%)	5 (8.2%)	NS
**HP infection**	12 (19.4%)	12 (19.7%)	NS
**Time to first diagnosis (day)**	202 ± 232	182 ± 210	0.62
**Body mass index (kg/m**^**2**^**)**	21.73 ± 4.48	20.99 ± 4.45	0.59
**Diagnosis circumstances**			
**Hematemesis**	0	12 (19.7%)	**0.001**
**Melena**	0	6 (9.8%)	**0.03**
**Anemia**	1	9 (14.8%)	**0.02**
**Weight loss**	33 (53.2%)	31 (50.8%)	0.79
**Dysphagia**	26 (41.9%)	29 (47.5%)	0.73
**Clinical signs**			
**Epigastric mass**	7 (11.3)	4 (6.6)	0.655
**Hepatomegaly**	1	2	0.836
**Ascites**	0	0	
**Troisier’s node**	0	1	

As predisposing factors for gastric cancer, smoking, and gastric ulcer are more common in the NSRCC group but with no statistically significant differences, *Helicobacter pylori* (HP) affected as many patients in both groups (19%).

Clinically, the consultation time was greater in the SRCC group, 202 vs 182 days with a nonsignificant *p*. The BMI was comparable in both groups. In the SRCC group, high digestive bleeding was more frequent (19.7% vs 0%; *p* = 0.001), as well as the presence of an epigastric mass (11.3% vs 6.6%; *p* = 0.655).

Concerning the macroscopic aspect of the tumor (Table 2), the budding aspect was much more seen in the non-SRCC group (78.7% vs 58.1%, *p* = 0.039) contrary to the infiltrative aspect which was much more seen in the SRCC group but without a significant difference (30.6% vs 14.8%, *p* = 0.060). Linitis plastica was almost exclusively seen in the SRCC group (22.6% vs 1.6%, *p* = 0.001). Moreover, there was no significant difference in tumor localization.

**Table 2:** Endoscopic characteristics

	SRCC group (***n*** = 62)	Non-SRCC group (***n*** = 61)	***p***
**Endoscopic aspect**			
**Ulcer**	51 (82.3%)	53 (86.9%)	0.53
**Budden**	36 (58.1%)	48 (78.7%)	**0.03**
**Infiltrative**	19 (30.6%)	9 (14.8%)	0.06
**Polypoid**	2	2	0.61
**Stenosing**	8 (12.9%)	6 (9.8%)	0.51
**Linitis plastica**	14 (22.6%)	1 (1.6)	**0.001**
**Tumor location**			
**Antral**	31 (50%)	36 (59%)	0.32
**Body**	28 (45.2%)	23 (37.7%)	0.44
**Small curvature**	23 (37.1%)	22 (36.1%)	0.59
**Large curvature**	9 (14.5%)	11 (18%)	0.51

Among the patients who underwent surgery (Table 3), there was no difference between the two groups in terms of ASA and WHO class. No patient had undergone neoadjuvant treatment because during the period of sudy, we had a problem of chemotherapy availability, and the waiting time to start treatment was too long also.

**Table 3:** Pre- and per-operative data

	SRCC (***n*** = 46)	Non-SRCC (***n*** = 45)	***p***
**ASA score**			0.950
**1**	34 (73.9)	32 (71.1)	
**2**	9 (19.6)	10 (22.2)	
**3**	3 (6.5)	3 (6.7)	
**WHO score**			0.617
**1**	18 (39.1)	16 (35.6)	
**2**	16 (34.8)	19 (42.2)	
**v3**	12 (26.1)	9 (20)	
**Tumor size (mm)**	63.9 ± 29.4	68.4 ± 37.8	0.55
**Distal metastasis**			
**Hepatic**	0	4 (8.9%)	**0.039**
**Other**	0	2	0.15
**Local organ invasion**	6 (13.3)	4 (8.7)	
**Carcinomatosis**	2	1	0.57
**Gastrectomy**			
**Subtotal**	6 (13%)	20 (44%)	**0.001**
**Total**	40 (87%)	25 (56%)	
**Lymph node dissection**			
**D1**	2	4 (9%)	0.38
**D2**	44 (96%)	41 (91%)	
**Quality of dissection**			
**R0**	36 (78%)	38 (84%)	0.063
**R1**	7 (15%)	1	
**R2**	3 (7%)	6 (13.3%)	

As for secondary localizations, hepatic metastasis was only seen in the non-SRCC group (9% vs 0%, *p* = 0.039), and also, local organ invasion was much more seen in the SRCC group (13.3% vs 8.7%, *p* = 0.479).

There were more SRCC patients in the total gastrectomy group 87% vs 55.6%, *p* < 0.001). Lymph node dissection was similar in the two groups. Besides, there was no significant difference regarding the excision quality, although R1 excision was much seen in the SRCC group (*p* = 0.063).

In terms of pathology (Table 4), there was no difference between the two groups. Yet in the SRCC group, tumors were classified as T3 or T4 in almost 60% of cases, N2 or N3 in almost 56% of cases, and stages II and III in 73% of cases.

**Table 4:** Histologic characteristics

	SRCC (***n*** = 46)	Non-SRCC (***n*** = 45)	***p***
**pT**			0.385
**T1**	4 (8.7)	8 (17.8)	
**T2**	14 (30.4)	10 (22.2)	
**T3**	17 (37)	15 (33.3)	
**T4**	11 (23.9)	10 (22.2)	
**pN**			0.073
**N0**	13 (28.3)	19 (42.2)	
**N1**	7 (15.2)	10 (22.2)	
**N2**	8 (17.4)	7 (15.6)	
**N3**	18 (39.1)	7 (15.6)	
**Stage**			0.112
**I**	11 (23.9)	12 (26.7)	
**II**	10 (21.7)	14 (31.1)	
**III**	24 (52.2)	14 (31.1)	
**IV**	1 (2.1)	5 (11.1)	

The overall mortality was null in the group SRCC, while it was 4.4% in the non-SRCC group, but there was no significant difference between the two groups (*p* = 0.148).

Patients in the non-SRCC group had significantly more medical complications than those in the SRCC group (17.8% vs 4.3%, *p* = 0.041).

There was no statistically significant difference between the two groups in terms of specific surgical complications (Table 5).

**Table 5:** Postoperative data

	SRCC (***n*** = 46)	Non-SRCC (***n*** = 45)	***p***
**Medical complications**	2 (4.3%)	8 (17.8%)	**0.04**
**Surgical complications**			
**Anastomotic leak**	3 (6.5%)	4 (8.9%)	0.672
**Duodenal leak**	2 (4%)	3 (6.7%)	0.627
**Hemorrhage**	0	0	
**Mortality**	0	2	0.148
**Recurrence**	8 (32%)	10 (35.7%)	0.77

There was no statistically significant difference between the SRCC and non-SRCC groups in the overall recurrence rate (32% and 35.7%, respectively, *p* = 0.776).

The most common type of recurrence in the SRCC group was peritoneal carcinosis, while the most common type of recurrence in the non-SRCC group was hepatic metastasis.

Overall 5-year survival in the SRCC group was lower than in the non-SRCC group, without a statistically significant difference (47.1 vs 51.5%; *p* = 0.715). There was also no significant difference in overall 5-year survival between the two groups, for both superficial and invasive cancer (Table 6).

**Table 6:** Recurrence and survival

	SRCC (***n*** = 34)	Non-SRCC (***n*** = 33)	***p***
**Tumor recurrence**	8 (32%)	10 (35.7%)	0.776
**Liver metastases**	1 (2.9%)	6 (60%)	0.096
**Peritoneal carcinosis**	5 (62.5%)	2 (6%)	
**Loco-regional**	2 (5.8%)	2 (6%)	
**5-year survival**	16 (47.1%)	17 (51.5%)	0.715
**Superficial cancer**	3 (100%)	4 (80%)	0.408
**Invasive cancer**	13 (41.9%)	13 (48.1%)	0.635

**Fig. 2:**
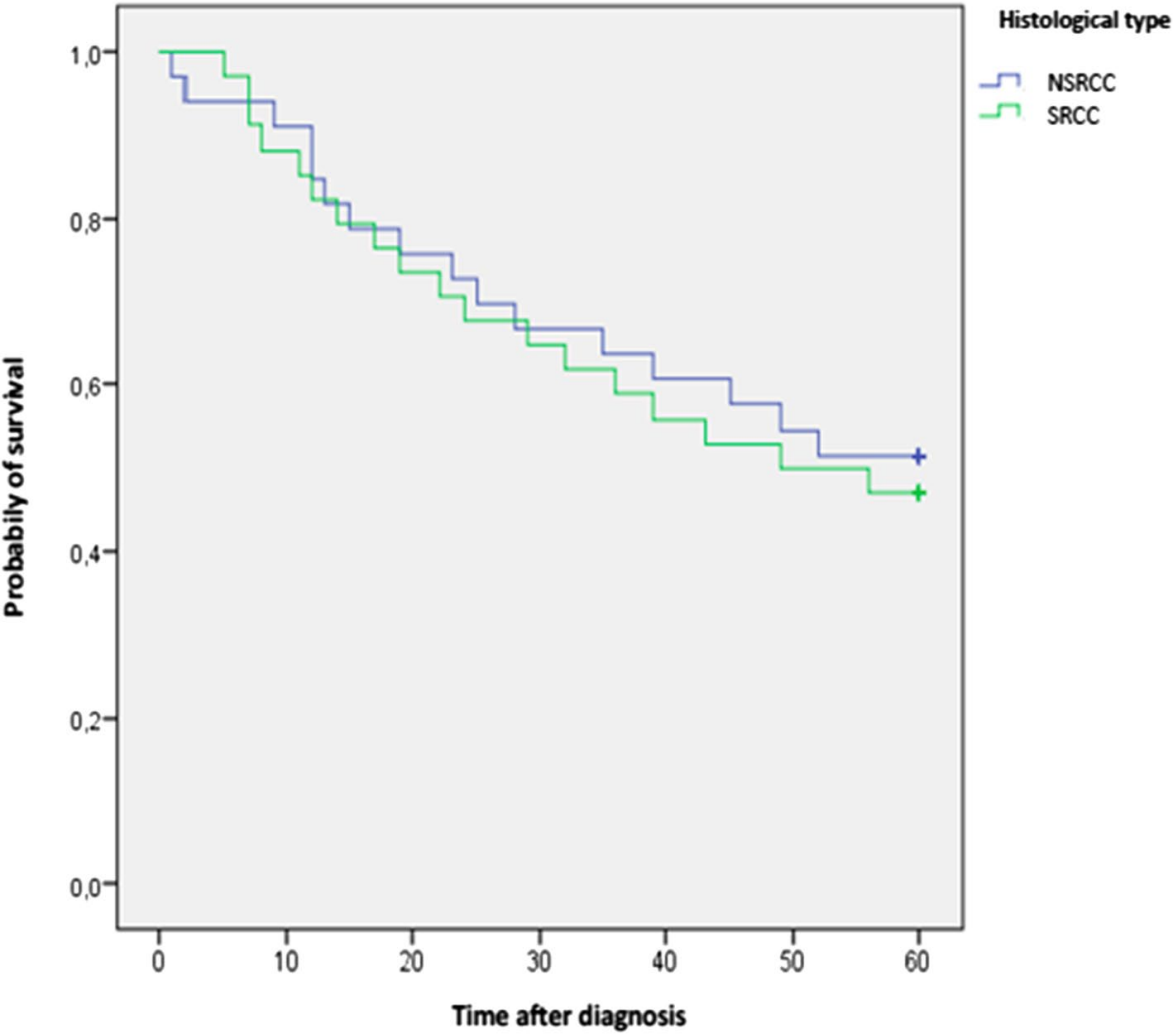
Overall survival according to the histotype

According to Kaplan-Meier survival curves (Fig. 2), we concluded that the overall survival was higher in the non-SRCC group but without a significant difference (47.1% vs 51.5%, *p* = 0.715).

Univariate cox regression analyses showed that malnutrition, linitis plastica, carcinomatosis, lymph node dissection type D1, palliative surgery, advanced tumor stage, and postoperative chemotherapy were associated with worse overall survival in the SRCC group (Table 7).

**Table 7:** Univariate cox regression analyses

	SRCC (***n*** = 34)			Non-SRCC (***n*** = 33)		
	***N***	5-year overall survival	***p***	***N***	5-year overall survival	***p***
**Age**			0.30			0.49
**≤ 60**	23	52.2%		10	60%	
**>60**	11	36.4%		23	47.8%	
**Sex**			0.49			0.31
**Female**	19	52.6%		14	64.3%	
**Male**	15	40%		19	42.1%	
**BMI**			0.82			**0.005**
**≤ 20**	5	40%		2	0%	
**>20**	7	42.9%		8	37.5%	
**Hemoglobin rate**			0.49			0.46
**≤ 10**	5	60%		19	47.5%	
**> 10**	24	41.7%		12	50%	
**Malnutrition**			**0.02**			0.67
**Yes**	2	0%		3	66.7%	
**No**	32	50%		30	50%	
**Tumor size**			0.09			0.47
**≤ 80 mm**	22	54.5%		22	59.1%	
**> 80%**	7	42.9%		9	33.3%	
**Linitis plastica**			**0.003**			-
**Yes**	7	14.3%		0		
**No**	27	55.6%		33	51.5%	
**Carcinomatosis**			**0.001**			-
**Yes**	2	0%		0		
**No**	32	50%		33	51.5%	
**Gastrectomy**			0.59			0.15
**Total**	29	44.8%		17	64.7%	
**Subtotal**	5	60%		16	37.5%	
**Lymph node dissection**			**0.001**			0.13
**D1**	2	0%		2	0%	
**D2**	32	50%		31	54.8%	
**Dissection quality**			**0.001**			**0.006**
**R0**	25	60%		28	60.7%	
**R1 + R2**	9	11.1%		5	0%	
**Lymph node involvement**			**0.001**			**0.002**
**Yes**	22	27.3%		17	29.4%	
**No**	12	83.3%		15	80%	
**Distal metastasis**						**0.02**
**Yes**	0	0		2	0%	
**No**	34	47.1%		31	54.8%	
**Tumor stage**			**0.003**			**0.01**
**I**	10	80%		10	80%	
**II**	8	62.5%		10	60%	
**III**	16	18.8%		11	27.3%	
**IV**	0	-		2	0%	
**Complications**			0.37			0.24
**Yes**	4	75%		10	40%	
**No**	30	43.3%		23	56.5%	
**Adjuvant chemotherapy**			**0.03**			0.21
**Yes**	20	30%		16	37.5%	
**No**	10	70%		13	69.2%	

Besides, concerning the non-SRCC group, *BMI* < 20 kg/m^2^, palliative surgery, advanced tumor stage, and distal metastasis were associated with worse overall survival (Table 7). Regarding multivariate cox regression analyses, lymph nodes involvement was an independent prognostic factor associated with worse overall survival for both groups SRCC and non-SRCC, whereas palliative surgery was an independent prognostic factor for the SRCC group only (Table 8).

**Table 8:** Multivariate analyses of prognostic factors

Group	***p***	***OR***	***CI*** 95%
**Non-SRCC**			
**Lymph node involvement**	**0.044**	4.073	1.038–15.982
**SRCC**			
**Dissection quality**	**0.026**	3.754	1.171–12.036
**Lymph node involvement**	**0.038**	30.097	1.208–749.987

## Discussion

SRCC incidence has been constantly increasing in Asia, the USA, and Europe accounting for 8 to 45% of cases of gastric adenocarcinoma [10, 17]. Its incidence increased tenfold between 1970 and 2000 [5].

In Tunisia, among the 503 patients with gastric cancer collected in the period going from January 2000 to December 2008, 173 patients (34.3%) had an SRCC [18]. In our series, the incidence of SRCC was equal to 50%.

SRCC is more common in female patients. It affects women more than non-SRCC with a sex ratio of 1 to less than 0.5. In Tunisia, there was a male predominance (sex ratio = 1.6). In our series, the sex ratio was 0.63 for SRCC against 2.05 for non-SRCC with a significant difference (*p* = 0.002).

It occurs in younger patients (7 years earlier than non-SRCC on average) with a mean age comprised between 55 and 61 [17, 19]. In Tunisia, the mean age at diagnosis was 56 [18]. In our series, the mean age of occurrence was 54.9 for SRCC against 62.5 for non-SRCC with a significant difference (*p* = 0.004).

In a study including more than 10,000 patients suffering from gastric cancer, SRCC was significantly more frequent in some ethnic groups such as Black and Asian people, Pacific Islanders, American Indians, Alaska Natives, and Hispanics [17].

There is no common risk factor between SRCC and non-SRCC. The latter is often multifactorial, whereas *Helicobacter* infection (HP) leading in most cases to chronic gastritis is implicated in most cases of gastric cancer without considering cancer of cardia.

Nevertheless, the role of HP in the occurrence of SRCC is a subject of much debate. As HP has been now widely eradicated, a new entity of gastric cancer occurring in HP-negative patients has emerged. This entity might include several subtypes such as adenocarcinoma of the fundic gland (GA-FG-CCP) and SRCC and thus call into question the role of HP in the occurrence of these subtypes [20].

In our study, there was no significant difference between SRCC and non-SRCC in terms of HP infection (19.4% and 19.7%, respectively, with *p* = 0.231), leading to the conclusion that it was not more implicated in the occurrence of SRCC than in that of non-SRCC.

The implication of other risk factors of gastric cancer (salty diet, smoking habit, autoimmune gastritis) in SRCC has not been well studied yet.

In our series, there was no significant difference between the SRCC and non-SRCC groups as far as the usual risk factors of gastric adenocarcinoma were concerned. Smoking habits, gastric ulcers, gastric polyps, Biermer’s disease, Menetrier’s disease, and partial gastrectomy did not seem to give more SRCC than non-SRCC.

The type of SRCC is strongly associated with mutations in the CDH1 gene which is responsible for the loss of the epithelial cell adhesion protein E-cadherin and for the loss of polarity of the gastric cell which becomes isolated. These mutations, whether germline or acquired, lead to the development of SRCC in the absence of any atrophic chronic gastritis and of intestinal metaplasia accounting for the fact that this histological type occurs in younger patients than other histologic types, which is confirmed by our study.

A hormonal theory according to which estrogens are implicated in the initiation or the tumoral progression or both has been put forward to account for the higher incidence of SRCC among women in comparison with non-SRCC. Our study also confirms these findings. Diffuse gastric cancer is more likely to present estrogen receptors even though this is not well-established in the subtype SRCC [21–23].

However, despite the suggestion that this mechanism might be implicated in the tumoral process, there is no evidence that it plays a major role.

Although gastric adenocarcinomas seem to present differently in Asian countries, and in Western countries [24–26], the five studies carried out in Europe and in the USA yield the same findings as those from Asian countries about the clinical presentation of SRCC [10, 17, 27–29].

SRCC is more commonly seen than non-SRCC in the median part of the stomach. In fact, contrary to gastric adenocarcinoma which is more commonly seen in the fundus of the cardia, SRCC has a marked preference for the antrum and the body of the stomach [30], which may result in a different clinical presentation. In our series, the tumor in both groups (SRCC and non-SRCC) was located in the order of frequency in the antrum, in the body, in the lesser curvature, and the greater curvature of the stomach, without a significant difference.

Another interesting finding revealed by the present study is as follows: symptoms associated with upper gastrointestinal bleeding (hematemesis, melena, iron-deficiency anemia) were significantly more frequent in the non-SRCC group and very rarely observed in the SRCC group.

A tumoral syndrome is more commonly observed for it is associated with more cancers in an advanced stage, and it is more frequent in stages IV, T3/T4, and N2 cancers [31].

In our series, a clinical tumoral syndrome was more frequently observed in the SRCC group with 11.3% of palpable hard masses against 6.6% in the non-SRCC group, without a significant difference (*p* = 0.655).

In addition, a Krukenberg’s tumor was diagnosed in 13.2% of patients in our series of the SRCC group, but none was found in the non-SRCC group. Anyway, the difference was not significant (*p* = 0.099).

In our series, SRCC had an ulcerative aspect in 82.3% of cases, a budding aspect in 58.1% of cases, and was infiltrative in 30.6% of cases. As for non-SRCC, it presented an ulcerative aspect in 86.9% of cases, a budding aspect in 78.7% of cases, and was infiltrative in 14.8% of cases.

Thus, the budding aspect was significantly more frequent in non-SRCC. The infiltrative characteristic was more frequent in the SRCC group, but the difference was not significant (*p* = 0.060).

Besides, linitis plastica was diagnosed in 22.6% of our patients suffering from SRCC against only one patient with non-SRCC. The difference was then significant (*p* = 0.001).

On one hand, the question of endoscopic resection is considered when the tumor is diagnosed early. Thus, Tong et al. have confirmed the importance of endoscopic resection in cases of SRCC diagnosed in an early stage due to their better prognosis [32].

In Europe and the USA, the consensus among the international experts of EORTC of St. Gallen is that endoscopic resections of superficial gastric cancer should largely depend on JGCA recommendations, except for diffuse gastric cancers in which cases surgery is indispensable [33].

Thus, in Western countries, endoscopic resection should not be performed in case of superficial SRCC whatever the degree of involvement of the gastric wall.

In our series, no endoscopic resection was performed in the patients with SRCC or non-SRCC either by resection of gastric mucosa or by DSM which is a procedure that is not commonly practiced in Tunisia. Moreover, as endoscopic resection is only considered after echo-endoscopy, easier access to this investigation procedure could increase the number of endoscopic resections of superficial gastric cancers in our country (Tunisia).

On another hand, due to the aggressiveness of SRCC, as soon as you cross the mucosal barrier, total preventive gastrectomy is usually recommended in patients aged between 20 and 30 years old who are healthy but have undergone a CDH1 gene mutation and have a family history of hereditary diffuse gastric cancer [30]. Family history particularly the age of onset of clinical manifestations should be taken into consideration in probands.

Despite an increased gastric lymph nodes involvement in SRCC, there is no specific recommendation as to the type of lymph nodes curettage to be performed in case of invasive SRCC. As for the other histological types, a modified D2 lymph nodes curettage consisting of the removal of at least 15 lymph nodes is recommended. This procedure was performed in 95.7% of cases of SRCC in our series.

As for distal gastric cancer, only two randomized clinical trials have focused on whether subtotal gastrectomy is sufficient in comparison with total gastrectomy. Both trials revealed no statistically significant difference in terms of death rate or survival rate between the two surgical procedures. We do not have at our disposal of subgroup analysis studies to assess both techniques in terms of the histological type. Thus, subtotal gastrectomy is recommended in cases of cancer of pyloric antrum independently of the histologic type. However, given the infiltrative nature of SRCC which leads to the resection of more frequently invaded proximal and distal margins (20.3% vs 9% and 20.3% vs 4%) in Piessen [34], some authors recommend total gastrectomy in case of independent cells adenocarcinoma of pyloric antrum. In our case, total gastrectomy was performed in 87% of cases of SRCC against 55.6% in cases of non-SRCC with a significant difference (*p* = 0.001). The rate of palliative resections R1 or R2 was higher in the SRCC group (21.7% vs 15.6%), but this difference was not significant (*p* = 0.063).

None of the articles consulted stated that in the case of SRCC, prophylactic bilateral ovariectomy should be practiced.

After all, because of the high rate of peritoneal carcinosis (17%) discovered in the course of surgical treatment of invasive SRCC, some surgeons propose two alternatives of therapy of SRCC. Firstly, exploratory laparoscopy can be systematically practiced to detect a possible peritoneal carcinosis which consequently results in a different treatment option. Secondly, in case of a per-operative discovery of resectable peritoneal carcinosis, palliative resection is not recommended in case of invasive SRCC due to the unacceptably high risk of postoperative death rate which is threefold higher for this histologic subtype [35].

The rate of peritoneal carcinosis in resected patients was 4.3% among the SRCC group against 2.2% in the non-SRCC group, without a significant difference (*p* = 0.570). In all cases, it was a located peritoneal carcinosis that was resected.

In general, the specific treatment for SRCC is as follows:

- Is early surgical resection for localized gastric tumor. This is the only effective treatment at this stage [36].
- Is surgical resection for advanced gastric tumor. As neoadjuvant chemotherapy has no benefit for survival, surgery is the most effective treatment at this stage [37].

Hu [38], a recent large study, about 12,484 patients with SRCC showed that the median of cancer-specific survival was 21 months in surgical group vs 5 months in the nonsurgical group (*p* < 0.001).

The death rate was nil in the SRCC group against 4.4% in the non-SRCC group. The difference was not significant.

The patients with non-SRCC suffered significantly from more medical complications than those with SRCC (17.8% vs 4.3%). There was no significant difference between the SRCC group and the non-SRCC group in terms of specific surgical complications (17.7% vs 22.2%). Reported findings concerning the prognosis of the different histological types encountered in gastric cancers are contradictory and seem to be influenced by the histological classification used (Lauren’s classification [9] or the WHO classification [39]) and by the stage of stomach cancer (superficial or invasive).

Thus, while all the published articles agree that the outlook of diffuse gastric adenocarcinoma, according to Lauren’s classification, is bleak [40, 41], including SRCC based on the WHO classification, the prognostic value of the latter is still a matter for debate.

On one hand, based on published studies, the histological prognosis of SRCC seems to be better [42, 43] or worse [15, 44] than that of other types of stomach adenocarcinomas (non-SRCC). It can also be equivalent to that of the other types [13, 27, 45]. On another hand, some studies [46–48] report a potential chemotherapy resistance of SRCC, which may influence survival since perioperative chemotherapy constitutes the treatment of choice for all gastric adenocarcinomas.

Based on an analysis of a large multicenter study of 1799 patients with gastric cancer who had undergone surgery for curative purposes between January 1997 and March 2010 in 19 French centers, Veron et al. [49] confirm the bleak prognosis of the histological type of SRCC in terms of survival rate in univariate analysis (median survival was 26 months vs 51 months in case of non-SRCC; *p* < 0.001) but also in multivariate analysis (*HR* = 1.182; *p* = 0.041) after adjustment of the other principal risk factors of death which are denutrition, the aspect of linitis, radical surgery, intraparietal tumoral extension (stage pT), lymph nodes invasion (stage pN), and the presence of distal metastases.

Histologic characteristics of SRCC have for a long time been considered as lesions of poorer prognosis than gastric cancers [34]. Recently, Tagavhi et al. showed through the study of a large cohort of 10,246 patients with gastric adenocarcinomas that for the same stage, SRCC and non-SRCC have the same prognosis [17]. This large-size retrospective study does not unfortunately take into account the new classification of SRCC and does not specify the share of independent cells to be able to specify the actual prognosis of SRCC [15, 47, 50].

If independent cells are not known to be a bad prognostic factor, the study by Taghavi et al. has excluded the gastric tumors restricted to the mucosa due to an absence of data. In these tumors T1 or early gastric cancer, the presence of independent cells seems on the contrary to be a good prognostic factor [15, 42, 51, 52].

T1 tumors have, in this case, a depressing aspect, stage II a–c according to Paris endoscopic classification [53]. The study by Taghavi et al. does not identify the presence of independent cells in gastric tumors as a poor prognostic factor, but it emphasizes the tumoral greater aggressiveness and the higher proportion of advanced stage. An SRCC at an equal tumoral stage and with equivalent lymph nodes curettage will more frequently have a lymphatic involvement and an incomplete surgical resection than a gastric adenocarcinoma [17]. Although this study is biased by the absence of information on the number of independent cells, SRCC tumors are considered more aggressive gastric tumors and require according to some teams a specific treatment for this entity.

Thus, SRCC is a stomach cancer that has a similar and even better prognosis when diagnosed early, but it is more aggressive than gastric adenocarcinoma with a greater risk of lymph nodes involvement or carcinosis at an advanced stage [30].

## Conclusion

The epidemiologic differences observed, the type of affected patients, the mode of revelation, the tumoral differences between SRCC and other histological types of adenocarcinomas, the prognostic value of histology type SRCC on overall survival, and the genetic and molecular differences described between the different histological types of gastric adenocarcinomas support the idea that SRCC should be considered as a separate entity among gastric adenocarcinomas.

## Data Availability

The datasets generated and/or analyzed during the current study are not publicly available due to department policy but are available from the corresponding author on reasonable request.
